# A Non-Surgical Multimodal Approach to Severe Thoracic Adolescent Idiopathic Scoliosis Combining ScoliBrace and Scoliosis-Specific Rehabilitation Therapies: A Case Series

**DOI:** 10.3390/healthcare13131522

**Published:** 2025-06-26

**Authors:** Anthony Nalda, Rosemary Mirenzi, Nora-Lee Doueihi, Jeb McAviney

**Affiliations:** 1 Private Practice, Celebration, FL 34747, USA; drtnalda@celebrationchiropractic.com; 2 ScoliCare, Syndey, NSW 2217, Australia; noralee.doueihi@scolicare.com (N.-L.D.); jeb@scolicare.com (J.M.)

**Keywords:** adolescent idiopathic scoliosis, AIS, ScoliBrace, ScoliBalance, TLSO, spinal rehabilitation, PSSE, non-surgical, multimodal, mirror image

## Abstract

Background/Objectives: Adolescent Idiopathic Scoliosis (AIS) is a lateral curvature of the spine combined with rotation and associated postural changes. Curves are classified according to direction and the spinal region, with right thoracic curves being a common presentation. Curve magnitude is measured using Cobb angles on radiographs and is used to monitor curve progression, with one of the main aims of treatment being prevention of progression to surgical levels. Treatment options may include observation, physiotherapeutic scoliosis-specific exercises (PSSE), thoracolumbosacral orthotic (TLSO) bracing, or surgery and are dependent on curve magnitude, risk of progression, and patient goals. Methods: This case series includes five patients (four female and one male, mean age of 14.8 y) who received previous non-surgical treatment without success and had severe right thoracic AIS with an average Cobb angle measurement of 53.4°, involving spinal curve magnitudes that warrant surgical recommendation. Results: These patients’ curves were successfully reduced to nonsurgical levels utilizing a non-surgical, multimodal treatment approach combining 3D corrective TLSO bracing using the ScoliBrace^®^, PSSEs, and spinal rehabilitation over an average of 37.0 months. The average Cobb angle reduced from 53.4° to 29.6° (44.6% reduction) after being weaned off treatment. Conclusions: This series has shown successful, clinically significant improvement in Cobb angle and trunk symmetry in five patients with severe AIS using a non-surgical, multimodal approach combining 3D corrective TLSO bracing using the ScoliBrace^®^ and spinal rehabilitation procedures. Further investigation into this multimodal non-surgical approach for children, parents, and healthcare providers and policymakers seeking an alternative to surgical intervention for AIS is warranted.

## 1. Introduction

Adolescent Idiopathic Scoliosis (AIS) is defined as a lateral curvature of the spine in the coronal plane measuring greater than a 10° Cobb angle on X-ray [1], accompanied by vertebral rotation and trunk deformity [2,3], and presenting between 10 and 17 years of age [4]. The condition has no determined direct underlying cause and is prevalent in approximately 2–3% of the adolescent population, with females affected more than males [5,6]. Curves tend to present and progress during the pubertal period of rapid growth [4,7,8].

Untreated AIS can lead to increased back pain and pulmonary symptoms for patients with large thoracic curves [5]. These patients can also develop substantial deformity [5]. Early-onset scoliosis is rarer yet has more severe consequences than AIS if left untreated [9]. These patients are more likely to progress to more severe curves and thoracic insufficiency, increasing the need for surgery, which is not without risks [10]. In adults living with AIS, it seems that quality of life and disability scores are increasingly correlated with decisions to have surgery when compared to the Cobb angle [11]. Some authors have also reported significant limitations in social activity due to back deformity [12,13,14]. Poor self-image scores compared to age-matched controls have also been reported [11,15]. Psychological disturbance has also been noted in 39% of women with thoracic curves larger than 40° [11]. Clinical features of AIS include spinal curvature, uneven shoulder height, asymmetrical scapula positioning, waistlines, and rib cages; translation or shift of the trunk in relation to the sacrum; and possible changes in kyphosis and lordosis in the sagittal plane [16].

AIS treatment decisions are often dependent on the risk of progression and are based on the size and location of the curve and remaining growth of the adolescent [4,5,17]. While remaining growth is a risk factor for progression, it also provides an opportunity for the growth modulation of the curve through treatment [18,19,20]. One of the primary considerations in improving scoliosis outcomes is that of aesthetic appearance [4,21,22,23], which may influence self-image and function [24].

Non-surgical treatment approaches for AIS include physiotherapeutic scoliosis-specific exercises (PSSE) for curves under 25° and PSSE combined with bracing for curves between 25 and 60° [4,17,25,26,27]. The aims of non-surgical management include preventing curve progression and the need for surgery [4,17,28,29], as well as improved aesthetics, quality of life, prevention of back pain, psychological well-being, and prevention of progression in adulthood [4,23]. Clinical studies have shown that the non-surgical management of AIS can reduce the prevalence of surgical intervention [30,31] and result in successful treatment outcomes [32,33] in curves below a surgical threshold of 45°.

Surgery is often recommended for patients whose curve is greater than 45° while still growing or continuing to progress beyond 45° when growth has stopped [34]. Surgical interventions include spinal fusion using metal implants to correct the spine and stabilize it in the improved position [34]. Many patients in surgical range reject surgical intervention for a multitude of reasons, including but not limited to an inability for spinal fusion to correct the signs and symptoms of scoliosis, complications of spinal fusion surgery including loss of normal spinal function, post-surgical pain, and continued curvature progression, as well as the prospect of needing revision surgery [35,36].

The aim of this case series is to present the results of a non-surgical, multimodal treatment approach for five surgical-level AIS cases, having attempted previous traditional non-surgical treatment, including various combinations of treatments with Boston braces, physical therapy, and chiropractic care. The multimodal treatment prescribed included a custom ScoliBrace^®^ spinal orthosis, worn 21 h per day, ScoliBalance^®^, CLEAR™ manual therapy techniques, and a daily, one-hour, home exercise program. The measurement of initial Cobb angles, the Angle of Trunk Rotation (ATR), coronal balance, and Trunk Aesthetic Clinical Evaluation (TRACE) scores were compared to post-treatment measurements.

## 2. Materials and Methods

### 2.1. Patient Inclusion Criteria

This case series reports on five patients who reported to a spinal rehabilitation facility with AIS. From 1 September 2016 to 30 September 2021, a total of 15 cases were identified to fit the predefined inclusion and exclusion criteria. Of these, five cases were selected for further analysis. For all patients, the following was consistent at their initial visit:

1. Health histories revealed right thoracic scoliosis diagnosed in the adolescent stage of development (AIS) (International Classification of Diseases, Tenth Revision (ICD-10) M41.12).
2. Posteroanterior (P-A) radiographic exams revealed severe (>45°) [4,23] right thoracic AIS Cobb angle measurements that meet surgical recommendation guidelines [4,23].
3. Standing posture assessment revealed right coronal imbalance, a right thoracic prominence, and uneven shoulders on all five patients.
4. Adam’s Forward Bend Test and ATR assessment revealed significant trunk rotation with an average of 12.4° right thoracic (ranging from 7° to 17° right thoracic).
5. TRACE revealed an average score of 8.6 (ranging from 8 to 9) out of a maximal score of 12, indicating trunk asymmetrical aesthetic changes.
6. Patients had re-exam and follow-up exam documentation to compare with initial examination findings and were compliant with treatment recommendations.

With regard to bracing duration and at-home scoliosis-specific exercise, compliance and adherence to the program were self-reported by the patients.

### 2.2. Patient Exclusion Criteria

Patients were excluded from the case series if any of the following conditions were met:

1. The patient/parent/carer was unable to give informed consent for involvement in the case series.
2. The patient had an underlying neuromuscular, congenital, or additional factor complicating the curve.
3. The patient was engaged in external or additional treatment programs.

### 2.3. Health Measures

#### 2.3.1. Radiographic Analysis

P-A spinal radiographs were taken at pre-treatment, ScoliBace^®^ fitting (in-brace), and post-treatment exams with the patient in a neutral, upright, weight-bearing position, allowing the detection of alignment abnormalities (i.e., rotations and translations of the head, spine, and pelvis from a normal position in a 3D coordinate system) [37].

The radiographs of the patient were examined using the Cobb method of measurement [38]. Coronal balance was measured using the distance between the C7 plumb line and the central sacral vertical line (CSVL) [39]. Coronal decompensation is usually defined as the 20 mm lateral deviation of C7 from the central sacral line in the frontal plane [40]. The Scoliosis Research Society (SRS) defines “compensation” as the vertical alignment of the midpoint of C7 within 15 mm of the sacral midpoint in the coronal plane [40].

An improvement in scoliosis has been defined conventionally as a reduction of the Cobb angle on X-ray of more than 5° [41,42,43]. Additionally, treatment is often aimed beyond solely reducing the Cobb angle [44] and incorporates other aims, including improving aesthetics, and reducing the risk to breathing function [23]. Reduction to under 15 mm in coronal balance has been determined by the SRS as an improvement when reporting outcomes of surgery for AIS but not specifically for non-surgical management [40]. For the purpose of this paper, the same measurement was used.

#### 2.3.2. Standing Posture Assessment

A P-A posture assessment was performed at pre-treatment, ScoliBace^®^ fitting (in-brace), and post-treatment exams using photography with the patient in a neutral, upright, weight-bearing position in front of a posture grid, allowing the detection of alignment abnormalities (i.e., rotations and translations of the head, thorax, and pelvis from a normal position in a 3-dimensional coordinate system) [37].

#### 2.3.3. Adam’s Forward Bending Test and Angle of Trunk Rotation (ATR)

Part of the AIS clinical examination is carried out by means of an Adam’s Forward Bend Test, which is often accompanied by scoliometer readings of the ATR, both of which have shown inter-examiner reliability [45]. This has become one of the main screening tools used for AIS [46], with a progression or improvement in ATR being identified by a minimum of a 2° change [41,42].

ATR is measured using a scoliometer run along the patient’s spine during an Adam’s Forward Bend Test. This is useful in determining the highest amount of trunk rotation in each region of the spine [47] to provide some indication of the three-dimensional deformity. Stable ATR measurements have been shown to be a safe, non-invasive, and cost-effective alternative to serial radiographs in the clinical monitoring of AIS [47]. It is not designed to be a diagnostic tool. Instead, it provides an indication of the need for an X-ray [48]. Coelho et al. (2013) noted the correlation between the scoliometer measurement and the X-ray measurement (r = 0.7 with *p* < 0.05) [49]. The main thoracic and thoracolumbar regions appear to be the most differentiating in the diagnosis of scoliosis [50]. It is possible to identify 87% of patients with a Cobb angle of 10° or more and 100% of the patients with curves greater than 20° using 5° ATR as the criteria for referral [49]. Bunnell recommended a minimum of 7° ATR as a criterion for referral to decrease the number of false positives [51].

#### 2.3.4. Trunk Aesthetic Clinical Evaluation (TRACE)

The TRACE tool is an objective four-item scale that evaluates four sub-scales: shoulders (0 to 3), scapulae (0 to 2), hemithorax (0 to 2), and waist (0 to 4), where 0 is no trunk asymmetry and the highest number, 12, is maximum trunk asymmetry [22]. This tool is sometimes used in clinical practice [52], providing individual scores for all aforementioned features and an overall global score to assess trunk aesthetics [53]. TRACE has proven to be a simple tool to implement in practice, with a change of 3 points or more out of 12 representing a significant change during treatment when the pre- and post-treatment observer is the same (intra-rater reliability) [54]. The TRACE scoring in the cases presented was conducted by a qualified physical therapist (RM) specializing in PSSE with more than seven years’ experience in using TRACE for AIS in day-to-day practice. The inter-rater reliability of the TRACE is currently considered poor, and thus reassessment was only performed by one assessor.

### 2.4. Patients’ Presentations

#### 2.4.1. Patient 1 (R)

A 15-year-old female (179.1 cm height, 51.8 kg) was diagnosed with a right thoracic AIS with a Cobb angle from T5 to L1 and apex at T9 (Cobb angle T5-L1 (T9)) measuring 37° on 9 June 2016. Reassessment by her surgeon on the 31 May 2017 showed progression of her Cobb angle to 50°, and surgery was recommended. The patient underwent a presurgical MRI on 21 September 2017, which showed no other pathology, and was scheduled for surgery on 3 September 2018.

On 4 February 2018, the patient presented to a spinal rehabilitation clinic and underwent postural and P-A full spine radiography examination. Pre-treatment P-A full spine stress radiographs showed curve flexibility [55]. The patient had a pre-treatment right thoracic ATR measuring 17° at T9 (0° is ideal), a right thoracic Cobb angle T4-L1 (T9) measuring 49° (0° is ideal), a TRACE score of 9/12 (shoulders 3, scapulae 2, hemithorax 2, and waist 2; 0 is ideal), and a Risser score of 3 (Table 1). The non-surgical multimodal treatment approach outlined in Appendix A Table A1 was recommended.

#### 2.4.2. Patient 2 (N)

A 15-year-old male (160.0 cm height, 43.1 kg) reported a family history of severe scoliosis and was diagnosed with right thoracic AIS with a Cobb angle T5-Tll (T9) measuring 15° in August 2015. The patient stated his doctor recommended he “watch and wait and return in a year.” At the one-year follow-up exam in August 2016, the patient stated his curve had progressed to 33°, at which time his doctor recommended a TLSO Boston Brace. The patient stated he decided to seek alternative options.

In November 2016, the patient and their mother presented to a spinal rehabilitation clinic and underwent postural and P-A full spine radiography examinations. Pre-treatment P-A full spine stress radiographs show curve flexibility [55]. The patient had a pre-treatment right thoracic ATR measuring 12° at T9 (0° is ideal), a right thoracic Cobb angle T5-T11 (T9) measuring 39° (0° is ideal), a TRACE score of 9/12 (shoulders 2, scapulae 2, hemithorax 2, and waist 3; 0 is ideal), and a Risser score of 0 (Table 1). The non-surgical multimodal treatment approach outlined in Appendix A Table A1 was recommended; however, the patient’s mother chose traditional chiropractic adjustments for 90 days. In January 2017, the patient returned to the spinal rehabilitation clinic to pursue the non-surgical multimodal treatment approach, at which point his Cobb angle increased to 48°.

#### 2.4.3. Patient 3 (B)

A 16-year-old female (171.5 cm height, 63 kg) was diagnosed with a right thoracic AIS with a Cobb angle T4-L1 (T8) in 2012 by her chiropractor. When the curvature started to show signs of rapid progression, she was referred to a spinal rehabilitation clinic.

On 26 February 2018, the patient presented to a spinal rehabilitation clinic and underwent postural and P-A full spine radiography examination. Pre-treatment P-A full spine stress radiographs showed curve flexibility [55]. The patient had a pre-treatment right thoracic ATR measuring 20° at T8 (0° is ideal), a right thoracic Cobb angle T4-L1 (T8) measuring 55° (0° is ideal), a TRACE score of 8/12 (shoulders 2, scapulae 2, hemithorax 2, and waist 2; 0 is ideal), and a Risser score of 4 (Table 1). The non-surgical multimodal treatment approach outlined in Appendix A Table A1 was recommended.

#### 2.4.4. Patient 4 (K)

A 13-year-old female (156.2 cm height, 43.5 kg) was diagnosed with a right thoracic AIS with a Cobb angle T5-L1 (T9) in 2018 by her chiropractor. When the curvature started to show signs of progression, she was referred to a spinal rehabilitation clinic in October 2018.

In October 2018, the patient presented to a spinal rehabilitation clinic and underwent postural and P-A full spine radiography examination. Pre-treatment P-A full spine stress radiographs showed curve flexibility [55]. The patient had a pre-treatment right thoracic ATR measuring 20° at T8 (0° is ideal), a right thoracic Cobb angle T5-L1 (T9) measuring 60° (0° is ideal), a TRACE score of 8/12 (shoulders 3, scapulae 1, hemithorax 2, and waist 2; 0 is ideal), and a Risser score of 0 (Table 1). The non-surgical multimodal treatment approach outlined in Appendix A Table A1 was recommended.

#### 2.4.5. Patient 5 (J)

A 15-year-old female (163.8 cm height, 57.2 kg) was diagnosed with a right thoracic AIS with a Cobb angle T5–T12 (T9) in 2017 by her chiropractor of 14 years. When the curvature started to show signs of rapid progression, she was referred to a spinal rehabilitation clinic in April 2019.

In April 2019, the patient presented to a spinal rehabilitation clinic and underwent postural and P-A full spine radiography examinations. Pre-treatment P-A full spine stress radiographs show curve flexibility [55]. The patient had a pre-treatment right thoracic ATR measuring 16° at T9 (0° is ideal), a right thoracic Cobb angle T5–T12 (T9) measuring 55° (0° is ideal), a TRACE score of 9/12 (shoulders 3, scapulae 2, hemithorax 2, and waist 2; 0 is ideal), and a Risser score of 3 (Table 1). The non-surgical multimodal treatment approach outlined in Appendix A Table A1 was recommended.

### 2.5. Non-Surgical Multimodal Spinal Rehabilitation

#### 2.5.1. ScoliBrace^®^ 3-D Over-Corrective TLSO Bracing

ScoliBrace^®^ is a customized, rigid, over-corrective thoraco-lumbo-sacral orthosis designed to place the patient in an in-brace position that attempts to correct the spine and posture in three dimensions using a Mirror Image^®^ approach [56,57,58,59]. Each ScoliBrace^®^ is custom designed and made for the individual patient with the latest in 3-D scanning technology and computer-aided design and manufacture (CAD CAM) (Canfit, V17), using a specific design algorithm and patient-centered approach to bracing treatment [57,60]. ScoliBrace^®^ uses an over-corrective approach to guide the body into a posture that is the opposite (Mirror Image^®^) to the way the scoliosis has positioned it, with the aim of reducing the Cobb angle where possible through axial elongation and counter-stress pressures. By putting the body posture in this over-corrected position, it directs the spine to begin to straighten using the concept of spinal coupling. As the body moves into the opposite position, the spine moves with it towards that position, achieving the maximum straightening within the limits of the spine and overall patient flexibility [58,61]. The patients were prescribed to wear this orthosis full-time (20–23 h per day) for improved treatment outcomes [62], as bracing dose has been shown to affect outcomes [63,64].

#### 2.5.2. ScoliBalance^®^ PSSE Program

The patients were instructed using ScoliBalance^®^ [58] as a PSSE program [4].

ScoliBalance^®^ is designed to help restore postural balance in the spine and trunk, maintain the integrity of balance and proprioception in the entire person, and reduce the Cobb angle of the curve if possible or prevent its progression in severe curves [58]. The program has been described elsewhere [58] and incorporates a combination of physiotherapeutic, chiropractic, and exercise rehabilitation principles and methods aimed at achieving the best possible postural correction for each patient.

#### 2.5.3. Chiropractic Leadership, Educational Advancement, and Research (CLEAR™) Scoliosis Institute Treatment

**Figure A1:**
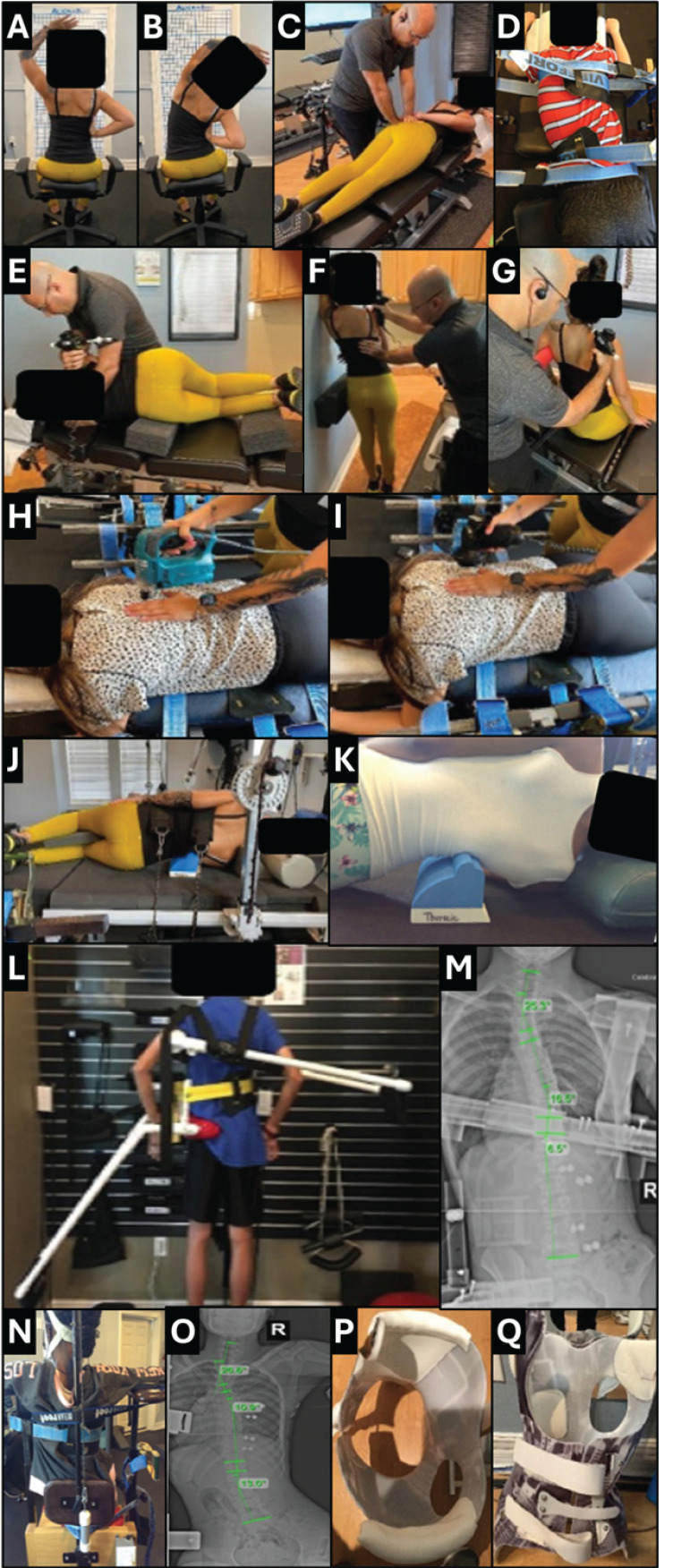
Non-Surgical, Multimodal Treatment Therapies for Right Thoracic AIS. (**A**,**B**) Wobble Chair Exercises; (**C**) Thoracic Mechanical Drop Adjustment; (**D**) Flexion/Distraction Table with Scoliosis Straps; (**E**–**G**) Mirror Image^®^ Adjusting; (**H**,**I**) Pettibon Instrument Adjusting; (**J**) Right Thoracic ScoliRoll^®^ Traction on Denneroll^TM^ Table; (**K**) Right Thoracic ScoliRoll^®^ Traction on Floor; (**L**) Total Body Weighting and Torso Trainer; (**M**) P-A Stress Radiograph Using Total Body Weighting and Torso Trainer; (**N**) Scoliosis Traction Chair; (**O**) P-A Stress Radiograph Using Scoliosis Traction Chair; (**P**,**Q**) ScoliBrace^®^ 3-D Corrective TLSO Brace.

The CLEAR™ Scoliosis Treatment Protocol is designed to address multiple factors related to the scoliosis presentation simultaneously [65]. It includes scoliosis-specific physical therapy exercises, specialized adjustments, and balance training exercises [65]. The details of this approach are in Appendix A Table A1 and Figure A1.

## 3. Results

### 3.1. Post-Treatment Exams

Re-evaluations occurred two weeks following the pre-treatment examination and then every 90 days thereafter. Active treatment lasted approximately 18 months, with an intensive two-week protocol every 6 months. Brace modifications and new braces were prescribed as needed due to reduction in Cobb angle, changes in curve shape, postural changes, and physical growth. After approximately 18 months of care, patients were weaned from treatment as desired treatment outcomes were achieved, defined by a lack of curve progression or improvement in curve magnitude. Post-treatment exams were performed at a mean of 37.0 months including health measures obtained at pre-treatment exams.

#### 3.1.1. Patient 1 (R)

Patient 1 was treated for 40.3 months using the non-surgical multi-modal treatment approach (Appendix A Table A1, Figure A1). At the initial ScoliBrace^®^ fitting (1 week after the initial consultation), the in-brace A-P full spine radiograph showed correction of Cobb angle T4-L1 (T9) measuring 10° (79.6% reduction). Reassessment on 10 May 2018 showed improved coronal balance in posture. Patient 1 was fitted with a new ScoliBrace^®^ TLSO in April 2019 due to skeletal growth and curvature improvement. At 40.3 months post-treatment, an A-P full spine radiograph showed improvement in the Cobb angle T4-L1 (T9) to 29° (40.8% reduction), removing her from the recommended Cobb angle measurement range for surgical treatment. Post-treatment ATR improved to 4° (76.5% reduction), indicating improvement in trunk symmetry. The TRACE score improved to 4/12 (55.6% improvement; Table 1). 

#### 3.1.2. Patient 2 (N)

Patient 2 was treated for 45.7 months using the non-surgical multi-modal treatment approach (Appendix A Table A1, Figure A1). At the initial ScoliBrace^®^ fitting, the in-brace A-P full spine radiograph showed correction of Cobb angle T5–T11 (T9) measuring 12° (75.0% reduction). Patient 2 was fitted with a new ScoliBrace^®^ TLSO in February 2018 and again in April 2020 due to skeletal growth and curvature improvement. Reassessment showed improved coronal balance in posture. At 45.7 months post-treatment, an A-P full spine radiograph showed improvement in the Cobb angle T5–T11 (T9) to 24° (50.0% reduction), removing him from the recommended Cobb angle measurement range for surgical treatment. Post-treatment ATR improved to 1° (91.7% reduction), indicating improvement in trunk symmetry. The TRACE score improved to 3/12 (66.7% improvement; Table 1).

#### 3.1.3. Patient 3 (B)

Patient 3 was treated for 39.9 months using the non-surgical multi-modal treatment approach (Appendix A Table A1, Figure A1). At the initial ScoliBrace^®^ fitting, the in-brace A-P full spine radiograph showed correction of Cobb angle T4-L1 (T8) measuring 20° (63.6% reduction). Patient 3 required ScoliBrace^®^ modifications in August 2018 and was fitted with a new ScoliBrace^®^ TLSO in September 2019, September 2020, and May 2021 due to skeletal growth and curvature improvement. Reassessment showed improved coronal balance in posture. At 39.9 months post-treatment, an A-P full spine radiograph showed improvement in the Cobb angle T4-L1 (T8) to 31° (43.6% reduction), removing her from the recommended Cobb angle measurement range for surgical treatment. Post-treatment ATR improved to 13° (35% reduction), indicating improvement in trunk symmetry. The TRACE score improved to 4/12 (50.0% improvement; Table 1).

#### 3.1.4. Patient 4 (K)

Patient 4 was treated for 32.7 months using the non-surgical multi-modal treatment approach (Appendix A Table A1, Figure A1). At the initial ScoliBrace^®^ fitting, the in-brace A-P full spine radiograph showed correction of Cobb angle T4-L1 (T8) measuring 21° (65.0% reduction). Patient 4 was fitted with a new ScoliBrace^®^ TLSO in June 2019 and again in December 2019 due to skeletal growth and curvature improvement. Reassessment showed improved coronal balance in posture. At 32.7 months post-treatment, an A-P full spine radiograph showed improvement in the Cobb angle T4-L1 (T8) to 34° (43.3% reduction), removing her from the recommended Cobb angle measurement range for surgical treatment. Post-treatment ATR improved to 2° (90% reduction), indicating improvement in trunk symmetry. The TRACE score improved to 4/12 (50.0% improvement; Table 1).

#### 3.1.5. Patient 5 (J)

Patient 5 was treated for 26.3 months using the non-surgical multi-modal treatment approach (Appendix A Table A1, Figure A1). At the initial ScoliBrace^®^ fitting, the in-brace A-P full spine radiograph showed correction of Cobb angle T5-T12 (T9) measuring 17° (69.1% reduction). Patient 5 was fitted with a new ScoliBrace^®^ TLSO in June 2021 due to skeletal growth and curvature improvement. Reassessment showed improved coronal balance in posture. At 26.3 months post-treatment, an A-P full spine radiograph showed improvement in the Cobb angle T5–T12 (T9) to 30° (45.5% reduction), removing her from the recommended Cobb angle measurement range for surgical treatment. Post-treatment ATR improved to 12° (25% reduction), indicating improvement in trunk symmetry. The TRACE score improved to 4/12 (55.6% improvement; Table 1).

It is important to note that all five patients were compliant with the clinician’s recommendations throughout the duration of the treatment protocol.

## 4. Discussion

This case series documents the first recorded successful improvement in the AIS Cobb angle and trunk symmetry of five AIS patients from a surgical to non-surgical recommendation status using a non-surgical, multimodal approach combining ScoliBrace^®^ TLSO, ScoliBalance^®^ PSSEs, and CLEAR™ therapies. Mean values showed an improvement of the Cobb angle from 53.4° to 29.6° (44.6%), right thoracic ATR from 17° to 6.4° (62.3%), and TRACE score from 8.6 to 3.8 out of a potential 12 (55.8%) in 5 patients (4 female and 1 male). This case series shows significant improvement from a surgical- to non-surgical-level Cobb angle in patients who were treated with the aforementioned multimodal approach. Significant improvements were also found in postural coronal balance, aesthetics (TRACE scores), and ATR. This treatment approach is likely to have worked due to the intensive and comprehensive approach to the treatment. It is also worth noting that the patients commenced treatment beyond the typically recommended Risser sign of 0–2 because the patients refused surgical treatment despite a surgical recommendation [4].

The ScoliBrace^®^ was prescribed to deliver a 3D, over-corrective, Mirror-Image^®^ approach for each patient [58], likely contributing to the outcomes. For larger curves, pairing it with ScoliBalance^®^—a PSSE program using a similar 3D, over-corrective method [58]—and CLEAR™ techniques may have increased brace comfort despite significant structural changes. This may have potentially improved compliance, a key factor in non-surgical AIS treatment success [64]. The ScoliBalance^®^ program strengthened the corrected curve posture, aiding condition stability as the patient reached growth completion. It also retrained balance, crucial for coronally imbalanced patients at treatment onset.

The influence of psychological motivation through regular contact with the treating clinician over the long-term follow-up should also be considered. With such significant improvements, it is also likely that patient compliance was positively affected by the promising results during the treatment process. All patients were compliant with the Clinician’s recommendations throughout the duration of the treatment protocol.

In non-surgical scoliosis management, “success” is defined as keeping the primary curve below 50° at skeletal maturity, with “failure” occurring otherwise [28,64]. This benchmark is problematic, as significant curve progression can still be labeled “successful” if under 50°—an outcome often unsatisfactory to patients and parents, driving demand for non-surgical options [66]. An “intensive” approach for AIS patients with 40–60° curves who rejected surgery, combining bracing and PSSE, achieved a 78% success rate [28]. Similarly, bracing alone in curves between 20 and 40° yielded a 72% success rate [64]. Unlike those studies, our “intensive” protocol involved a multimodal strategy delivered in two-week blocks over time until skeletal maturity, targeting curves exceeding surgical thresholds and larger than those previously reported. Our case series found that five patients with thoracic curves of 48–60° reduced to non-surgical levels with long-term follow-up—a notable outcome, given thoracic curves’ higher brace failure risk compared to lumbar curves, despite similar initial sizes and wear times [67].

For patients compliant with brace wear (>12.9 h/day, per the BrAIST study cut-off [64]), brace failure (surgery or progression to ≥50°) occurred in 30.3% of those with main thoracic curves versus 5.3% with main lumbar curves (*p* = 0.0239) [67]. Larger curves combined with lower Risser scores at brace initiation strongly predict failure [67]. Our research offers fresh insight, showing that patients who have not yet reached skeletal maturity with large thoracic curves, who decline surgery, can reduce Cobb angles to non-surgical levels using a multimodal approach.

This paper explores a non-surgical strategy for surgical-level curves, which are typically considered at 45–50°. Conventionally, bracing and PSSE are advised beforehand, but dosage beyond bracing hours is seldom considered. Our study introduces a more intensive approach for severe curves, demonstrating potential success by combining curve flexibility enhancement through CLEAR™ techniques and a PSSE program through ScoliBalance^®^, with ScoliBrace^®^.

This study’s limitations stem from its retrospective design and small sample of five patients, raising potential recall and selection bias. With only five patients and no control group, the study lacks the statistical power to draw robust conclusions regarding treatment effect. The limited population and sample size restrict the study’s applicability to other settings or patient groups (patients with different curve patterns, differing age groups, etc.). Patient-reported outcomes with regard to bracing and exercise compliance may be influenced by recall bias. Adherence to bracing or PSSE protocols likely varies among patients but is difficult to quantify retrospectively, confounding treatment effectiveness. Additionally, not all exposures, potential confounders, and effect modifiers were noted for all patients.

However, given the niche population—patients with surgical-level right thoracic curves seeking non-surgical options—this study approach appeared fitting. In addition, the use of standardized objective metrics (Cobb angle, ATR, etc.) allows for effective comparison between cases within the study. While research on bracing and PSSE for curves below 45–50° is expanding, studies on non-surgical management of surgical-level curves remain scarce.

Future research into non-surgical management for those rejecting surgery should involve larger cohorts. Randomization is often unattainable in this population; however, propensity score matching or stratification could control for confounding variables, including age, curve severity, or skeletal maturity. Incorporation of a control group would assist in the clarification of treatment effects. Collecting patient satisfaction and quality-of-life data would enable comparisons with outcomes from surgical or untreated patients.

## 5. Conclusions

This case documents the first observed significant improvement in AIS Cobb angle and trunk symmetry of five AIS patients from surgical to non-surgical recommendation status following participation in a conservative, non-surgical, multimodal approach combining ScoliBrace^®^ TLSO, ScoliBalance^®^ PSSEs, and CLEAR™ therapies. Right thoracic AIS patients (including those who have been recommended surgery) may benefit from an examination from spinal rehabilitation clinics that use ScoliBrace^®^ TLSO, ScoliBalance^®^ PSSEs, and CLEAR™ therapies to see if a non-surgical multimodal approach may be possible.

## Figures and Tables

**Table 1:** Pre-Treatment and Post-Treatment Cobb Angles, ATR, TRACE Scores, Risser Signs, and Treatment Duration for Patients 1–5.

Health Measures		Patient 1	Patient 2	Patient 3	Patient 4	Patient 5	Mean
Initial Age (y) Post-Treatment Age (y)		15 18	15 19	16 19	13 16	15 17	14.8 17.8
Sex		F	M	F	F	F	-
Height (cm)		179.1	160.0	171.5	156.2	163.8	166.1
Weight (kg)		51.8	43.1	63	43.5	57.2	51.7
Pre-Treatment Cobb Angle (°)		49	48	55	60	55	53.4
Post-Treatment Cobb Angle (°)		29	24	31	34	30	29.6
Δ Cobb Angle (°(%))		−20(40.8)	−24(50.0)	−24(43.6)	−26(43.3)	−25(45.5)	−23.8(44.6)
Pre-Treatment ATR (°)		17	12	20	20	16	17
Post Treatment ATR (°)		4	1	13	2	12	6.4
Δ ATR (°(%))		−13(76.5)	−11(91.7)	−7(35)	−18(90)	−4(25)	−10.6(62.3)
Pre-Treatment TRACE Score	Shoulder Score	3	2	2	3	3	2.6
	Scapulae Score	2	2	2	1	2	1.8
	Hemithorax Score	2	2	2	2	2	2
	Waist Score	2	3	2	2	2	2.2
	Overall Score	9	9	8	8	9	8.6
Post-Treatment TRACE Score	Shoulder Score	1	1	1	1	1	1
	Scapulae Score	1	0	1	1	1	0.8
	Hemithorax Score	1	1	1	1	1	1
	Waist Score	1	1	1	1	1	1
	Overall Score	4	3	4	4	4	3.8
Δ TRACE Score (n(%))		−5(55.6)	−6(66.7)	−4(50.0)	−4(50.0)	−5(55.6)	−4.8(55.8)
Pre-Treatment Risser Sign		3	0	4	0	3	2
Post-Treatment Risser Sign		5	5	5	4	4	4.6
Treatment Duration (months)		40.3	45.7	39.9	32.7	26.3	36.98

y = year, cm = centimeter, kg = kilogram, ° = degree, Δ = change, % = percent, ATR = angle of trunk rotation, TRACE = trunk aesthetic clinical evaluation, n = number.

## Data Availability

The original contributions presented in this study are included in the article. Further inquiries can be directed to the corresponding author.

## Abbreviations

The following abbreviations are used in this manuscript:

3-D	three-dimensional
AIS	adolescent idiopathic scoliosis
GBD	global burden of disease
YLD	years lived with disability
PSSE	physiotherapeutic scoliosis specific exercise
ICD-10	International classification of diseases, tenth revision
P-A	posterior-to-anterior
ATR	angle of trunk rotation
TRACE	trunk aesthetic clinical evaluation
S-I	superior-to-inferior
I-S	inferior-to-superior
cm	centimeter
kg	kilogram
°	degree
Δ	change
%	percent
n	number
CLEAR™	Chiropractic Leadership, Educational Advancement & Research

## Appendix A

**Table A1:** Non-Surgical, Multimodal Treatment Prescription for Right Thoracic AIS.

Therapy/Exercise	Repetitions/Duration	Description
Wobble Chair Exercises	50 repetitions	A wobble chair is used for exercises that challenge stability and reflexes to maintain an upright posture [68].
Exercises on Whole Body Vibration Plate	10 min	Strengthening exercises while being challenged on a whole body vibration plate [69].
Thoracic Mechanical Drop Piece	10 min	A technique used to mobilize a restricted or stiff joint in a very controlled manner [70].
Flexion/Distraction Table with Scoliosis Straps	20 min	A table designed specifically for scoliosis treatment with the aim of providing gentle, intermittent traction to the scoliotic spine [71].
Mirror Image^®^ Adjusting	Not applicable	Mirror Image^®^ adjutsting uses the phenomenon of how an object (spine/posture) can end up in a totally different three-dimensional orientation based on reversing the order of two or more sequential movements [61]
Pettibon Instrument Adjusting	10 min	A form of percussion therapy using Pettibon instruments with the aim of relaxing deep spinal muscles. This is performed asymmetrically [65].
Right Thoracic ScoliRoll^®^ Traction on Denneroll^TM^ Table	10 min	The ScoliRoll^®^ (Denneroll Industries, Sydney, AUS) is an orthotic traction device designed specifically for scoliosis patients [58,72]
Scoliosis Traction Chair	20 min	Use of gentle spinal traction to reduce scoliosis whilst the patient is in a seated position. Within this position, the patient’s spine is simultaneously de-rotated, elongated, and straightened [73].
Total Body Weighting and Torso Trainer	10 min	This method attempts retrain the internal postural mechanisms in the brain by inducing a reaction through added weights. The body learns to correct itself against the weight which becomes ingrained in the muscle memory and postural mechanisms over time [74].
ScoliBalance^®^ Exercises	40 repetitions	A physiotherapeutic scoliosis specific exercise (PSSE) program approach was used [58] that incorporated the PSSE components as recommended by international guidelines [4].
CLEAR Specific Scoliosis Exercises	10 repetitions	Individualised set of scoliosis-specific exercises prescribed based on three-dimensional X-ray analysis and physical examination [75]
ScoliBrace^®^ Corrective Brace with Exercises	Brace 21 h, Exercises 1 h	Every ScoliBrace^®^ is custom made for the individual by a dedicated design team and Computer Aided Design and Manufacturer. Unlike other traditional style scoliosis braces, which use a 3-point pressure system, ScoliBrace^®^ uses an inverse corrective approach that harnesses spinal coupling to maximise correction where possible. In most cases ScoliBrace^®^ uses an over-corrective, Mirror Image^®^ approach to guide the body into a posture that is the opposite of the way the scoliosis has positioned it. By putting the body posture in this over-corrected position, it forces the spine to straighten up using the concept of spinal coupling i.e., as the body moves into the opposite position, the spine moves with it towards that position achieving the maximum straightening within the limits of the spine’s flexibility [58].
